# Efficient oil/saltwater separation using a highly permeable and fouling-resistant all-inorganic nanocomposite membrane

**DOI:** 10.1007/s11356-020-08021-x

**Published:** 2020-02-19

**Authors:** Rand Elshorafa, Jayaprakash Saththasivam, Zhaoyang Liu, Said Ahzi

**Affiliations:** 1 Division of Sustainable Development, College of Science and Engineering, Hamad Bin Khalifa University, Education City, P.O. Box 5825, Doha, Qatar; 2 Qatar Environment and Energy Research Institute, Hamad Bin Khalifa University, Qatar Foundation, P.O. Box 5825, Doha, Qatar

**Keywords:** Membrane separation, Oily wastewater, Superoleophobic, Nanomaterials, Fouling resistance

## Abstract

Although it is still a great challenge, developing oil-/water-separating membranes that combine the advantages of high separation efficiency, salty environments tolerance, and fouling resistance are highly demanded for marine oil spill cleanups and oil-/gas-produced water treatment. Here, we report a new type of all-inorganic nanostructured membrane, which is composed of titanate nanofibers and SiO_2_ particulate gel for efficient and stable oil/saltwater separation. The nanoporous and interconnected network structure constructed with titanate nanofibers is the key to ensure the high separation efficiency and high water flux of the new membrane. The SiO_2_ gel is used as a binder to offer mechanical flexibility and integrity for this type of all-inorganic membrane. The new membrane displays a high oil/water separation efficiency of above 99.5% with oil content in treated effluent lower than US environmental discharge standards (42 ppm) and high water permeation flux of 1600 LMH/bar under low operation pressure. The new membrane also demonstrates outstanding durability in the environment of different salinities, and it has a good resistance for oil fouling due to its excellent underwater superoleophobicity with an oil contact angle above 150 °. Most importantly, the underwater superoleophobic properties can be well maintained after being repeatedly reused. The excellent environmental durability, oil-fouling resistance, high separation efficiency, and facile fabrication process for this new type of membrane render great potential for industrial application in treating produced water.

**Electronic supplementary material:**

The online version of this article (10.1007/s11356-020-08021-x) contains supplementary material, which is available to authorized users.

## Introduction

Oil pollution is a serious issue faced globally because of the large amounts of oily and salty wastewater caused by frequent marine oil spill accidents and oil exploitation/production/refinery activities (Qin et al. 2015; Wang et al. 2017a). Regulations enacted by the US Environmental Protection Agency (EPA) limit the discharge of oil in the effluents to a maximum of 42 mg/L (Anon 2010). Therefore, it is in great demand to develop effective and sustainable techniques to treat oil-polluted wastewater in order to satisfy the stringent regulation and preserve the environment. Conventional techniques for oil/water separation, such as the hydrocyclone and air floatation, suffer some drawbacks including either low separation efficiency or high operation cost (Qin et al. 2017; Wang et al. 2017b). Membrane techniques, such as microfiltration (MF), ultrafiltration (UF), and nanofiltration (NF), which operate with “size-sieving” effect, have been widely used for water purification (Zhang et al. 2013, 2014). UF membranes are very effective in separating stabilized oil emulsions and especially for removing oil droplets with sizes in the micrometer range to produce water with low oil concentrations. However, commercial UF membranes suffer from severe fouling or low fluxes which result from pore plugging by oil droplets and segregated pore structures, and this significantly limits their service time and degrades their separation performance in practical applications (Obaid et al. 2018; Shi et al. 2013).

Recently, superwetting membranes have been demonstrated for efficient oil/water separation by designing superhydrophobic–superoleophilic or superhydrophilic–superoleophobic surfaces in conjunction with surface chemistry and roughness (Zhu et al. 2014). For example, a Teflon (PTFE)-coated mesh film with superhydrophobicity was reported for the separation of oil and water mixtures (Feng et al. 2004). However, these hydrophobic membranes are easy to be fouled by oil, resulting in a quick decline of both water flux and separation efficiency. Therefore, superhydrophilic and superoleophobic membranes are more suitable for the separation of water-rich oil/water mixtures or emulsions. Under the inspiration of fish scales, high-energy materials with water-favoring property were proposed for the construction of underwater superoleophobic membrane surfaces. For example, hydrogel-coated mesh was fabricated with underwater superoleophobicity for separating oil/water mixtures and surfactant-stabilized emulsions (Xue et al. 2011; Zhang et al. 2018). However, these polymer-based approaches have weaknesses in their consideration of practical applications, including the stability of polymeric coating under severe conditions as well as long-term preservation for reuse (Sun et al. 2012). Until now, superoleophobic membranes for oil/water separation with high separation efficiency, superior oil-fouling resistance, and high environmental durability are still barely applied in industry.

In this study, we report a new all-inorganic nanocomposite membrane for oil/saltwater separation. The new membrane is composed of titanate nanofiber and silica nanoparticulate gel. The nanofiber-constructed network membrane presents high separation efficiency for oil/saltwater emulsions with oil residual in the filtrates lower than environmental discharge standards (42 ppm). The silica gel coating ensures the flexibility and integrity of this all-inorganic membrane. Meanwhile, the membrane shows excellent antifouling properties due to its underwater superoleophobicity. Most importantly, this all-inorganic membrane exhibits good durability under harsh environments without a decline in terms of both water flux and separation efficiency. Its excellent oil/water separation efficiency, durability, fouling resistance, and low-cost fabrication render this new all-inorganic nanocomposite membrane a great potential for practical industrial applications.

## Material and methods

### Materials

All the chemicals used in this study (titanium dioxide (P25)(TiO_2_), ethanol tetraethyl orthosilicate (TEOS) Si(OC_2_H_5_)_4_, ethanol (CH_3_CH_2_OH), ammonium hydroxide (NH_4_OH), n-hexane C_6_H_14_, and n-Octane C_8_H_18_ were purchased from Sigma Aldrich. In addition to that, DI water was used.

### Instruments and characterization

The concentration of oil in the feed and filtrate samples was analyzed by measuring the total organic carbon using TOC analyzer (Shimadzu, TOC-L, Japan). The oil droplet size and distribution in the feed was measured by using a Visual Process Analyzer (JORIN-ViPA). The surface morphology of the membrane was analyzed by using scanning electron microscopy (SEM, FEI Corp.), while X-ray diffraction instrument (XRD, Bruker D8 Advance, Bruker-AXS, Germany) was used to determine the crystal structure of the membrane materials. The hydrophilicity/hydrophobicity properties of the membrane were obtained by measuring the water contact angles by using an advanced goniometer (Rame-hart A100, USA).

### Materials preparation

#### Titanium nanofibers preparation

Titanium (Ti) nanofibers were prepared as per the procedure described by Jung et.al (2014). Firstly, 7.5 mg/mL of TiO_2_ nanoparticle (anatase P25) solution was prepared by adding 0.375 g of TiO_2_ nanoparticles to 50 ml of a10 M NaOH solution. The solution was well mixed for 30 min and then transferred into an autoclave where it was placed in a mechanical convection oven for 4 days at 220 °C. The hydrothermal reaction between TiO_2_ particles and NaOH resulted in the formation of titanate nanofiber hydrogels. The titanate nanofibers (TiNFs) were cooled at room temperature and washed multiple times using deionized water to neutralize the pH. The solution was then filtered, dried overnight, and stored at room temperature.

#### SiO_2_ nanoparticles preparation

In this work, hydrophilic SiO_2_ nanoparticles were synthesized by adopting the method used by Cai et.al (2014) where TEOS, ethanol, and ammonia were used as a precursor, a solvent, and a catalyst, respectively. Firstly, 1 mol of TEOS was mixed with 1 mol of ethanol for 30 min using a magnetic stirrer at room temperature. Then, 115.8 mol of water and 0.01 mol NH_3_ were added and mixed for 8 h using a magnetic stirrer at 40 °C. The resulting SNP solution was then cooled down to room temperature prior to the membrane deposition.

#### Free-standing TiNF/SiO_2_ membrane preparation

TiNF/SiO_2_ membrane was fabricated using a vacuum filtration apparatus, where a premixed solution that contained titanium nanofibers and hydrophilic SiO_2_ was deposited on a temporary cellulose substrate under a low-vacuum condition. The premixed solution was prepared by adding 0.08 g of TiNFs into 40 mL of deionized water and 10 mL of SiO_2_ nanoparticle solution. Prior to deposition, the solution was probe-sonicated for 5 min to homogenize the dispersion of the nanoparticles. The deposited nanoparticles were washed multiple times using deionized water and finally dried at room temperature for 24 h. Upon drying, the deposited TiNF/SiO_2_ membrane could be easily peeled out from the cellulose substrate.

#### Preparation of oil-in-water emulsion

A stable oil-in-water emulsion can be prepared using a sonication technique (Sakai 2008). Firstly, 1 mL of oil was mixed with 45 mL of deionized water and then sonicated by using an ultrasonic water bath for 5 min. The resulted emulsion was then further diluted with deionized water to a total volume of 100 ml (Kayvani et al. 2018) to achieve a final concentration of 1% *v*/v oil-in-water emulsion. In this study, oil-in-water emulsions were prepared using different types of oils, namely, sunflower oil, diesel, gasoline, octane, and hexane. Salinity in the emulsion feed was varied from 2500 ppm to 45,000 ppm by adding an appropriate amount of sodium chloride (Kayvani et al. 2018).

#### Performance test

The performance of the TiNF/SiO_2_ membrane was evaluated based on the oil rejection and the permeate flux. The performance test studies were conducted by using a vacuum filtration apparatus where the operating pressure was fixed at vacuum pressure of − 30 kPa.

#### Calculation procedure

##### Flux calculation

The flux through the membrane was demined using the following equation:

1 $$ Flux\left( Lmh/ bar\right)=\frac{V}{S\ t\ P} $$

where V is the volume of the permeated water, S is the surface area of the membrane, t is the testing time, and P is the applied pressure.

##### Oil rejection

The oil rejection will be calculated using the following equation:

2 $$ R\left(\%\right)=\left[1-\frac{C_p}{C_f}\right]x\ 100 $$

where R is the rejection percentage, C_f_ is the feed concentration, and C_p_ is the permeate concentration. A TOC analyzer was used to measure oil content in the filtrate and feed water samples.

## Results and discussions

### SEM

**Fig. 1:**
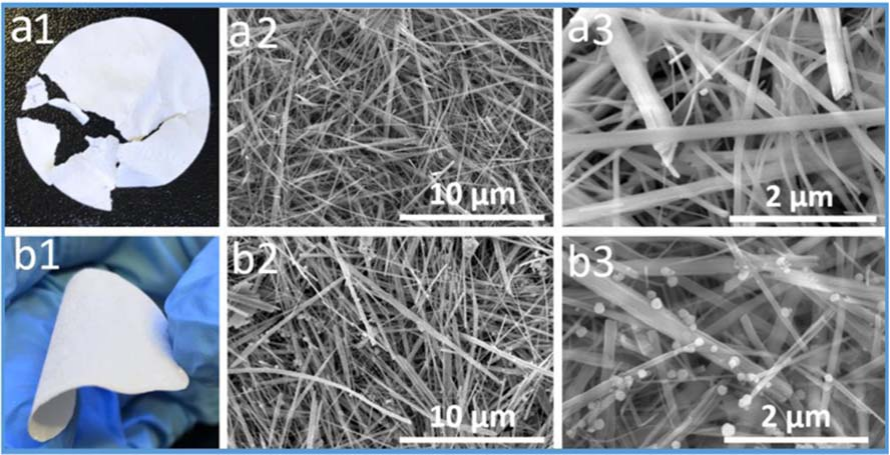
**a1** A photo of the TNF membrane made out of pure titanate nanofibers showing the fragility of the TNF membrane. **a2** An SEM image of the TNF membrane surface material showing the interconnected nanoporous structure. **a3** An SEM image with higher magnification of the TNF membrane surface. **b1** A photo of a free-standing TNFS membrane made of titanate nanofibers and silica nanoparticulate gel showing the flexibility of the TNFS membrane. **b2** An SEM image of the TNFS membrane surface showing the interconnected nanoporous structure. **b3** An SEM image with higher magnification of the TNFS membrane surface showing the titanate nanofibers bonding with the silica nanoparticulate gel

Two types of all-inorganic nanostructured membranes were prepared, one with pure titanate nanofibers (TNF) and another with titanate nanofiber/silica gel (TNFS), respectively. The photographic images in Fig. 1 highlight the differences between TNF and TNFS membranes. Figure 1 a1 shows the cracked TNF membrane made out of pure titanate nanofibers without the addition of silica gel. This TNF inorganic membrane is fragile. Figure 1 a2 and a3 show the interconnected nanostructure of the TNF membrane constructed with titanate nanofibers. The 3D interconnected pore structure and tight pore size could render the membrane with high water flux and high oil rejection. The length and the diameter of the fabricated TNFs are 20 μm and 100 nm, respectively. Figure 1 b1 shows the photo of the flexible free-standing TNFS membrane made out of titanate nanofibers and silica gel. When silica nanoparticulate gel was coated to TNF membranes, as seen in the SEM of Fig. 1 b3, the flexibility of the membrane was significantly enhanced. The silica nanoparticulate gel functions as an inorganic binder on titanate nanofibers. The flexibility of the membranes is very important during practical industrial applications.

### XRD and EDS

**Fig. 2:**
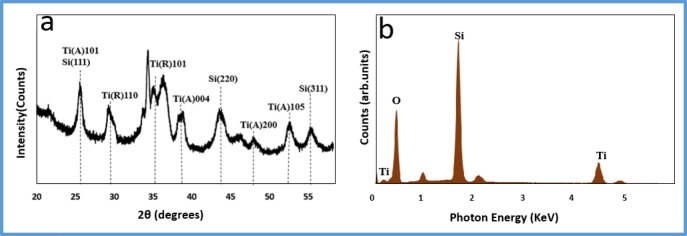
**a** An XRD spectra of the TNFS membrane. The major peaks indicate the presence of titanate and silica in the membrane. **b** An EDS analysis of the TNFS membrane

Figure 2 shows the XRD and the EDS analysis of the TNFS membrane. The obtained XRD profile of titanate and silica is in agreement with the findings of other literatures. The crystalline orientations of silica (111), (220), and (311) are clearly shown in Fig. 2a, which correspond to 2θ ° of 25.5 °, 43.9 °, and 55.6 °, respectively (Aceves-Mijares et al. 2012; Yasar-Inceoglu et al. 2012). The crystalline orientations which correspond to the anatase structure of the titanate are (101), (004), (200), and (105) which correspond to 2θ ° of 25.5 °, 38.9 °, 48.1 °, and 52.7 °, respectively (Yoon et al. 2017; Zhang et al. 2002). The crystalline orientations that correspond to the rutile structure of the titanate are (110) and (101) which correspond to 2θ ° of 29.5 °, and 35.1 °, respectively (He et al. 2015; Yoon et al. 2017). Figure 2b shows an EDS analysis of the TNFS membrane, which further confirms the presence of the silica binder coated on the TNF-based membrane.

### FT-IR

**Fig. 3:**
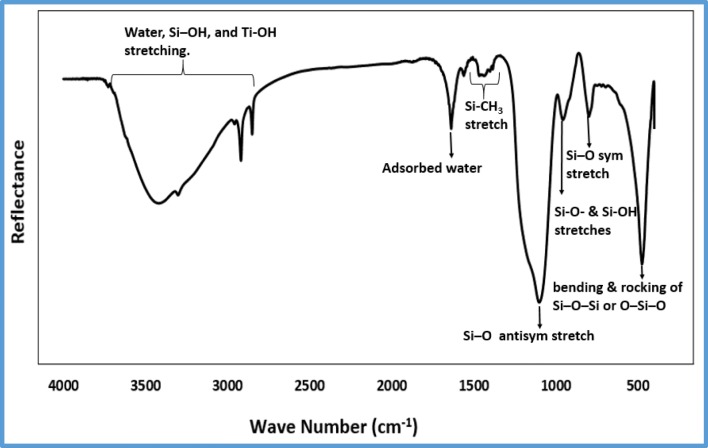
FT-IR spectra of the TNFS membrane, which indicates the good hydrophilicity of the TNFS membranes made of titanate nanofibers and silica gel

Figure 3 represents the FT-IR analysis of the TNFS membrane in the frequency range of 4000–550 cm^−1^. The purpose of the FT-IR analysis was to identify the functional groups on the material surfaces, which could provide the indication about hydrophilicity and hydrophobicity. This is because increasing the water concentration leads to a complete hydrolysis reaction of TEOS and the condensation reaction then forms a 2D linear chain and a 3D net structurer that contain a lot of (-OH) groups. The peaks at 1100 cm^−1^ and 796 cm^−1^ correspond to Si-O antisymmetric and symmetric stretches, respectively (Brusatin et al. 1997). The peak at 957 cm^−1^ corresponds to Si-O and Si-OH stretches (Rajput et al. 2012). The peak at 474 cm^−1^ corresponds to the bending and rocking of Si-O-Si or O-Si-O (Agarwal et al. 1995).The wide stretching band between 3700 cm^−1^and 2840 cm^−1^ corresponds to water, Si-OH, and Ti-OH stretching (Sun et al. 2017). The peak at 1640 cm^−1^ corresponds to adsorbed water (Sárkány 2002), which indicates the good hydrophilicity of the membrane surfaces.

### Membrane wettability

#### Contact angle

**Fig. 4:**
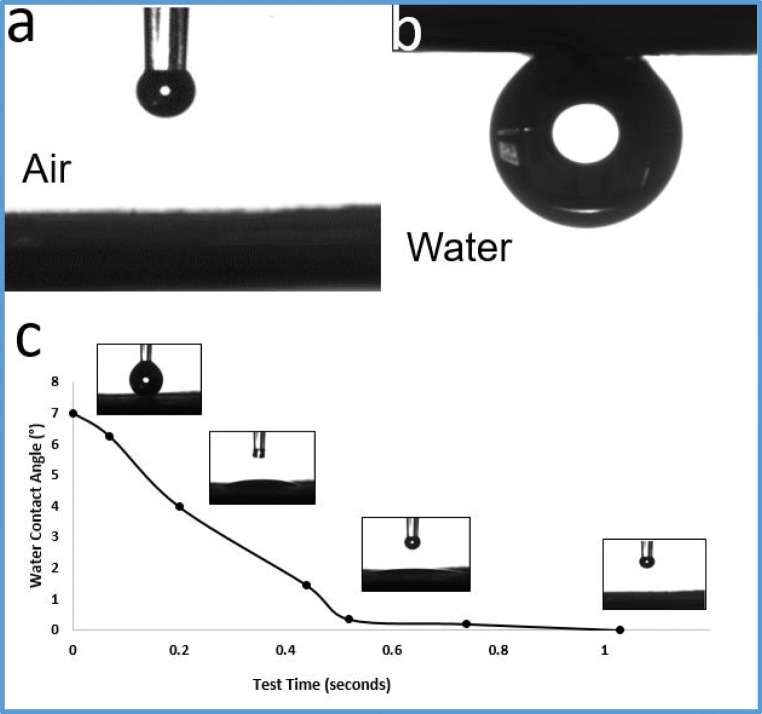
**a** A water contact angle of the TNFS membrane (0°). **b** An underwater oil contact angle of the TNFS membrane (156 ± 1.6°). **c** Dynamic water contact angle: water droplet behavior when water drops on the TNFS membrane

In order to assess the hydrophilicity of the membrane, it is important to perform a water contact angle (WCA) and an oil contact angle (OCA) (Sun et al. 2019; Yang et al. 2019a, b). The water droplet behavior was assisted by measuring the dynamic water contact angle when dropping the water on the TNFS membrane. As shown in Fig. 4, the contact angle measurements showed that the TNFS membrane has a water contact angle (WCA) of 0 °, indicating the superhydrophilic property of this membrane. On the other hand, the underwater oil contact angle (OCA) was 156 ± 1.6 °, which indicates the underwater superoleophobicity of the membrane. The superhydrophilic and underwater oil-repelling surface of the new all-inorganic membrane is crucial in minimizing fouling caused by oil and organic substances.

### Performance test

#### Digital images before and after membrane treatment

**Fig. 5:**
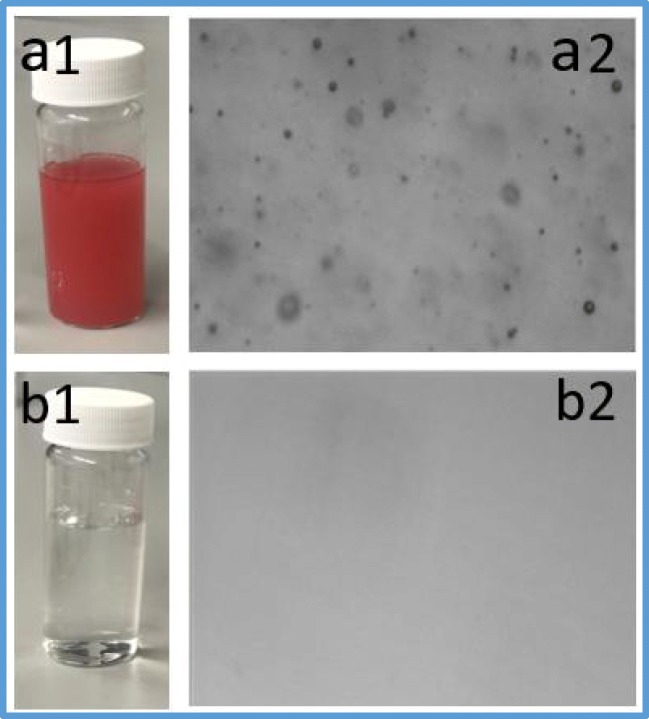
Photo images of oil-in-water emulsions before and after filtration by using the TNFS membranes. **a1** A photo of the feed solution before filtration, and the oil was dyed in red. **a2** A microscopic image of oil droplets in the feed solution before filtration. **b1** A photo of the permeate solution after filtration by using the TNFS membranes. **b2** A microscopic image of permeate solution after filtration, which shows that the solution becomes clear and that there are no visible oil droplets after filtration by using the TNFS membranes

Figure 5 shows the photo images of the emulsified oil droplets in the feed and permeate solutions. The emulsified oil was dyed in red for visualization purposes. Before filtration, the VIPA microscopic image shows the existence of oil droplets in the emulsion, as seen in Fig. 5 a2. After filtration, the VIPA microscopic image shows the absence of oil droplets in the permeate after filtration by using the TNFS membranes, as seen in Figure 5 b2. This is a direct indication that the emulsified oil has been completely removed by using the TNFS membranes.

#### Different oil

**Fig. 6:**
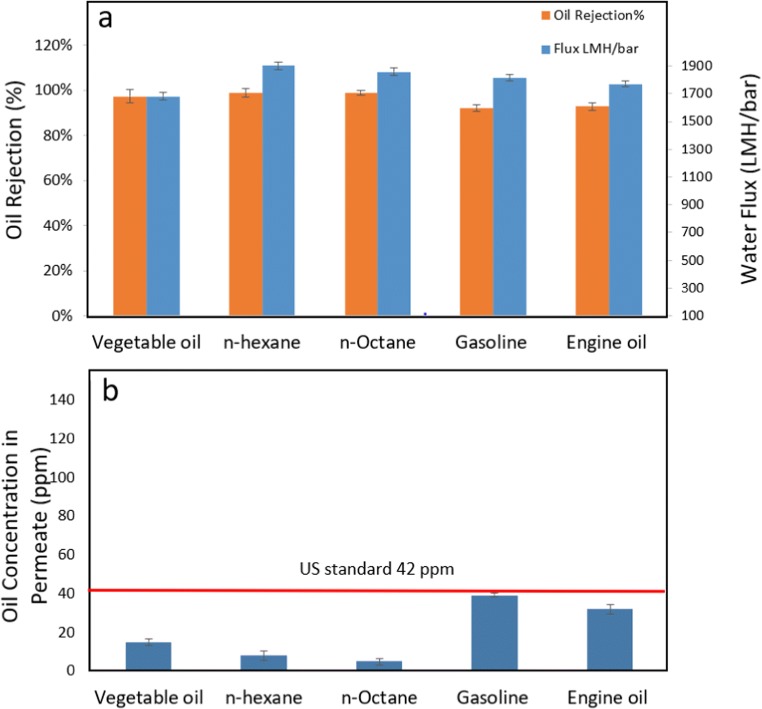
**a** Performance tests for the TNFS membranes in terms of oil rejection and water flux with different oils and **b** oil concentrations in permeates, which are lower than US environmental discharge standards of 42 ppm

Figure 6 shows the performance test results of the new TNFS membranes in terms of oil rejection and water flux. The different types of emulsified oil which include (i) vegetable oil (ii) n-hexane (iii) n-octene, (iv) gasoline, and (v) engine oil have been tested. The oil rejection is over 99% for all types of oils. It is worth to note that the oil concentrations in the permeates of the TNFS membranes are below the stringent oil discharge limit of 42 mg/L imposed by US EPA regulation. As shown in Fig. 6b, which indicates that it is safe to discharge the permeates to the environment, the high oil rejection rate could be due to the tight nanoporous structure of the new TNFS membranes.

According to Fig. 6a, the water fluxes ranged from 1681.4 LMH/bar to 1902.6 LMH/bar. The water flux of these TNFS membranes is significantly higher than the reported water flux for polymer and ceramic membranes (Fan et al. 2015; Hu et al. 2004; Kayvani et al. 2018). The high water flux could be due to its 3D interconnected pore structure, which is easy for water to pass through.

#### Salty environment tests

**Fig. 7:**
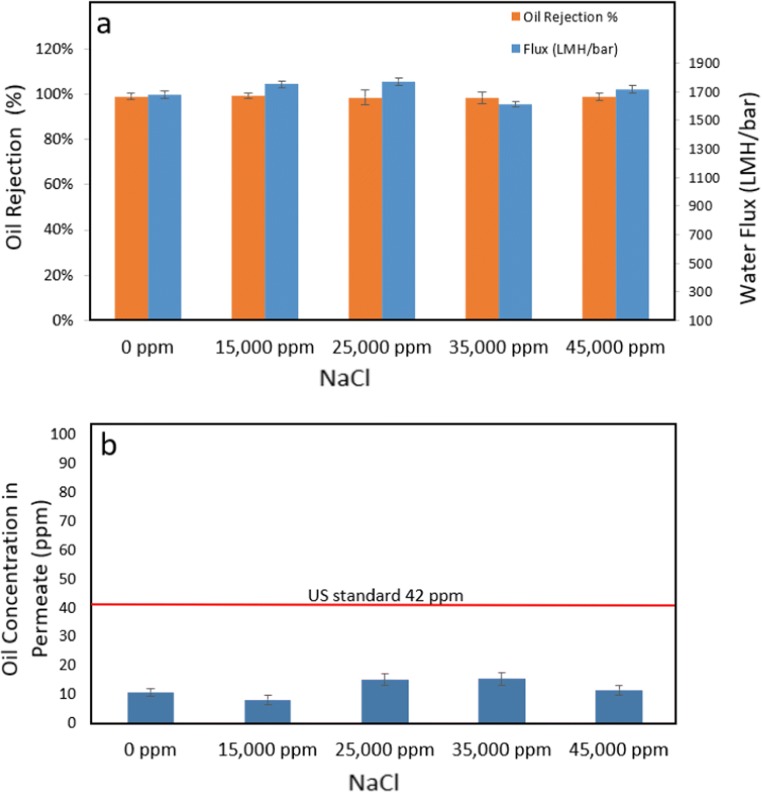
**a** Performance tests in terms of oil rejection and water flux under different salt concentrations and **b** oil concentrations in the permeates under different salt concentrations, which indicate that the TNFS membranes can effectively separate oil under different salty environments

It is important to test the membrane performance under different saline environments as oil-/gas-produced water and offshore oil spills which usually contain different levels of salinities. Figure 7 shows the performance test results of oil rejection and water flux under different salt concentrations. Figure 7a shows that the separation rates for the emulsified oil/water solutions can reach as high as 99% under different salinities. As shown in Fig. 7b, the oil concentrations in the permeates ranged between 8 ppm and 15 ppm, which is lower than US EPA regulation limits (42 ppm). As shown in Fig. 7a, the high salinities did not affect the performances of the new TNFS membranes in terms of oil rejection rate and water flux.

#### Reusability test

**Fig. 8:**
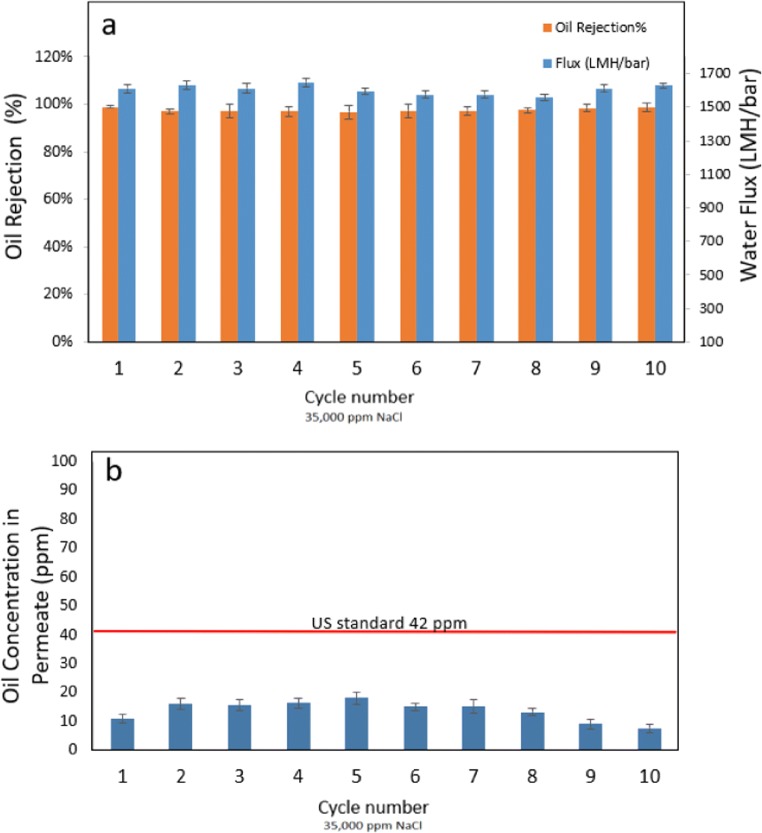
The reusability test of the TNFS membrane. **a** Oil rejection and water flux as a function of 10 cycles of reuses. **b** Oil concentration in the permeate as a function of 10 cycles of reuses

The reusability of the TNFS membranes was tested over a period of 10 filtration cycles. As shown in Fig. 8, oil rejection rates remain constant at around over 99% during all the 10 operating cycles. The oil contents in the permeate were consistently lower than the US EPA oil disposal limit (42 ppm).These results demonstrate the excellent durability of the TNFS nanocomposite membranes, as they are made out of all-inorganic materials. The superhydrophilic and oil-repelling surface of the TNFS membranes can be maintained consistently.

## Conclusions

In this study, a new type of all-inorganic nanostructured TNFS membrane was presented for efficient and stable oil/saltwater separation. The new membrane displays high oil/water separation efficiency of above 99.5% with oil content in treated effluent lower than US environmental discharge standards (42 ppm) and high water permeation flux of 1600 LMH/bar under low operation pressure. Most importantly, the underwater superoleophobic property can be well maintained after repeated reuses in the environment of different salinities. The excellent separation efficiency, durability, fouling resistance, and low-cost fabrication render this new membrane with great potential for treating produced water and offshore oil spills.

## Electronic supplementary material

ESM 1 (DOCX 3.64 mb)
