# Supplementary material for: Efficient oil/saltwater separation using a highly permeable and fouling-resistant all-inorganic nanocomposite membrane
Source: Environ Sci Pollut Res Int. 2020 Feb 19;27(13):15488–97. doi: 10.1007/s11356-020-08021-x (PMC7190607; doi:10.1007/s11356-020-08021-x)
Supplement: Supplementary file 1 — (DOCX 3.64 mb) [file 11356_2020_8021_MOESM1_ESM.docx]

**Supporting Information:**

**Efficient oil/saltwater separation using a highly-permeable and fouling-resistant all-inorganic nanocomposite membrane**

Rand Elshorafa, Jayaprakash Saththasivam, Zhaoyang Liu*, Said Ahzi*

Qatar Environment and Energy Research Institute, Hamad Bin Khalifa University, Qatar Foundation, PO Box 5825, Doha, Qatar

Corresponding: [zhliu@hbku.edu.qa](mailto:zhliu@hbku.edu.qa) ; [sahzi@hbku.edu.qa](mailto:sahzi@hbku.edu.qa)

**Membrane preparation procedure**


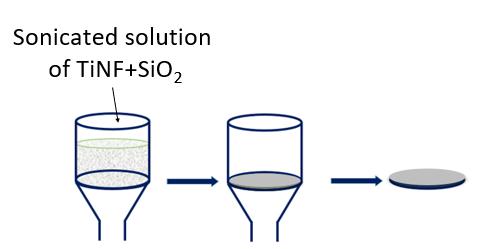


Figure S1: TNFS membrane preparation using vacuum filtration

**Excess SiO_2_**


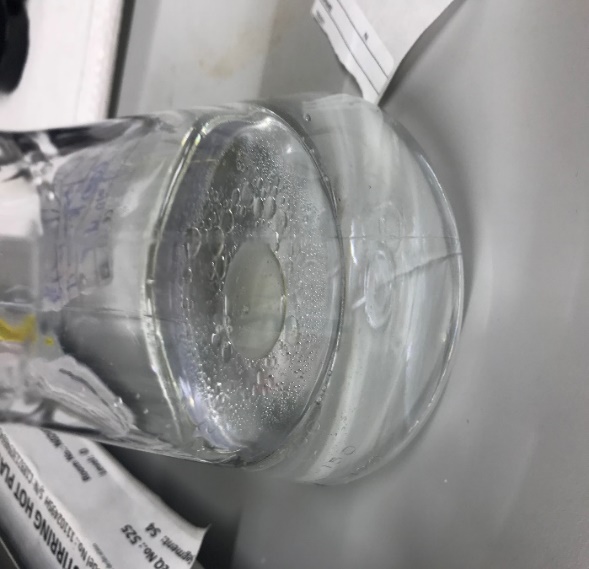

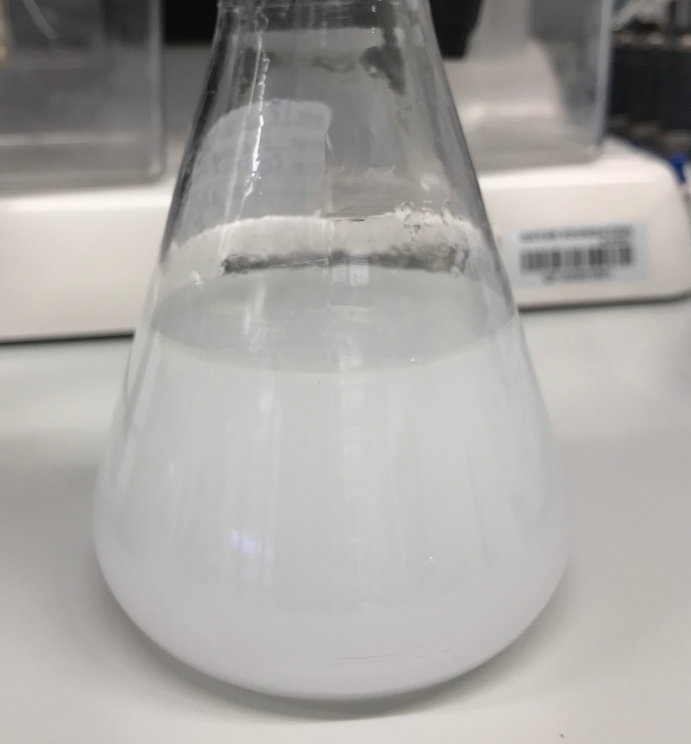

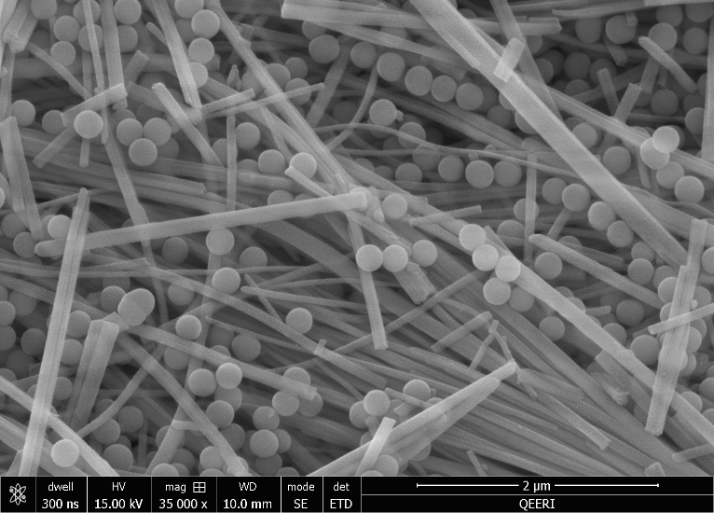


After sonication: one phase

Before sonication: Two phases

Figure S2: TNFS membrane with excessive silica gel coating.

**Vegetable oil size distribution**


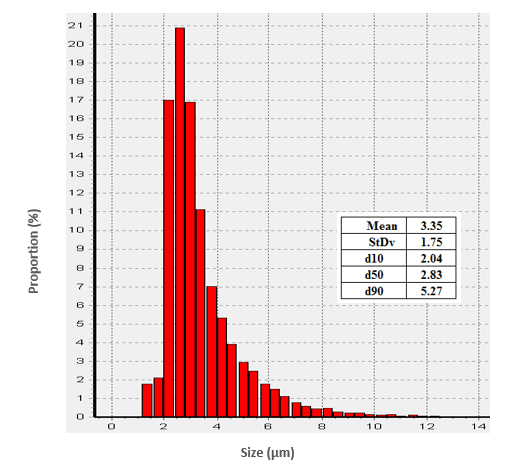


Figure S3: Emulsified vegetable oil size distribution in feed solutions.


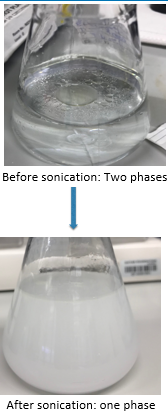


Figure S4. Photos of oily feed solutions before and after sonication for the emulsion preparation.

**Ethanol rinsing**

The membrane was rinsed with ethanol after performing the emulsion separation. Figure S5 shows the photo of the flexible free standing TNFS membrane after ethanol rinsing. Thus, ethanol rinsing did not effect the mechanical flixibility of the membrane. Figure S6 represents the SEM of the membrane after ethanol rinsing. As shown in figure S6, ethanol rinsing did not affect the nanostructure of the membrane. Figure S7 represents the dynamic water contact angle: water droplet behavior when water drops on the TNFS membrane after ethanol rinsing. The membrane contact angle is zero degrees after 1 second of droping the water drop. This indicates that the membrane weatability was not affected by ethanol rinsing. The reusability of the TNFS membranes was tested over a period of 5 filtration cycles using ethanol rinsing between each cycle. As shown in Figure S8, oil rejection rates ramain constant at around over 99%, during all of the 5 operating cycles. The oil contents in the permeate were consistently lower than the US EPA oil disposal limits (42 ppm).These results demonstrate the excellent durability of the TNFS nanocomposite membranes, as it is made out of all inorganic materials. The super-hydrophilic and oil-repelling surface of the TNFS membranes can be maintained consistantly.


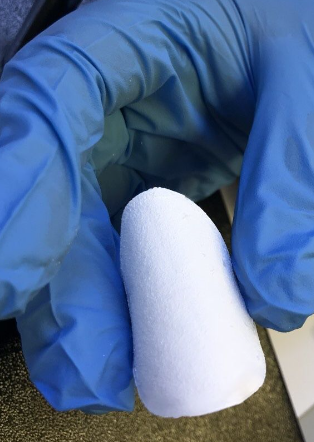


Figure S5. Photo of the membrane after ethanol rinsing.


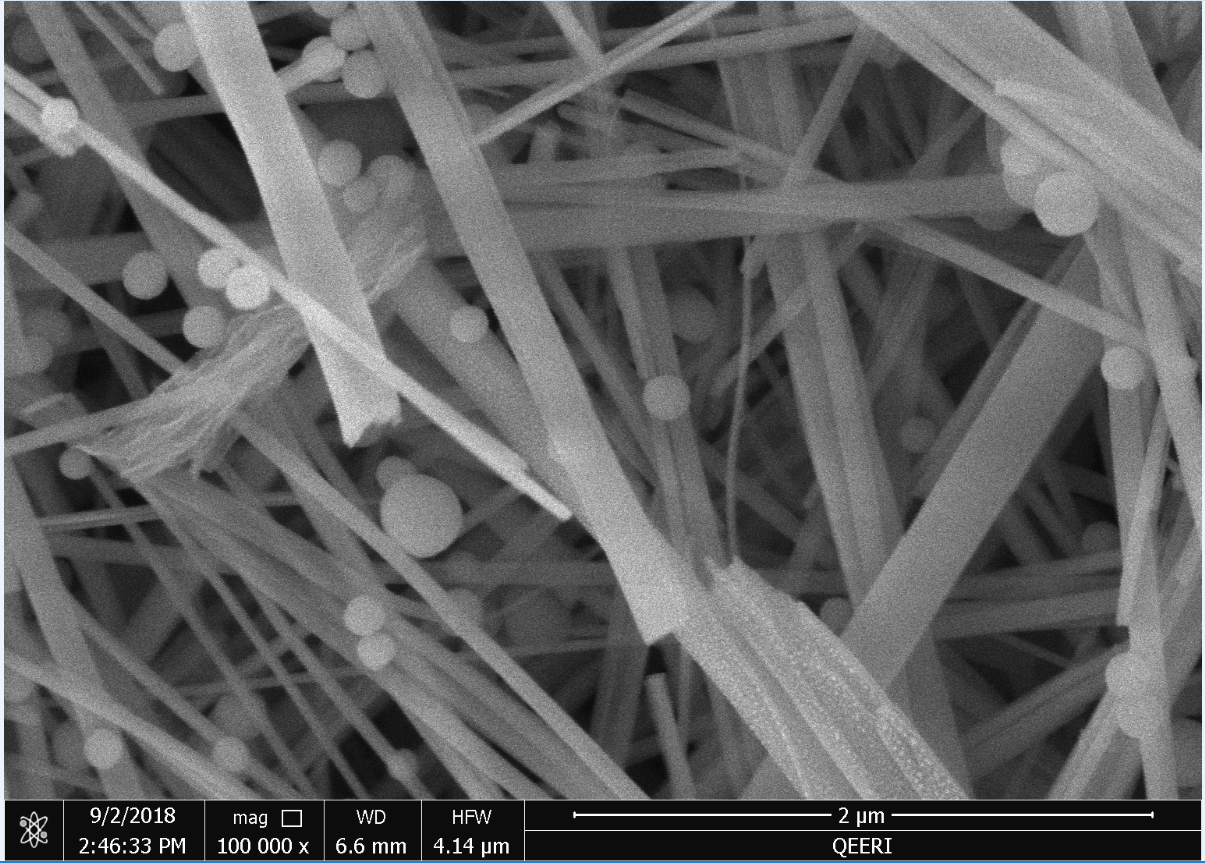


Figure S6. SEM of the membrane after ethanol rinsing.


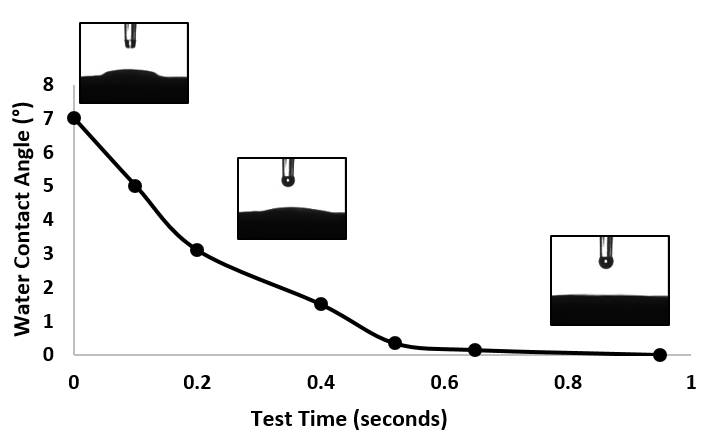


Figure S7. Dynamic water contact angle: water droplet behavior when water drops on the TNFS membrane.


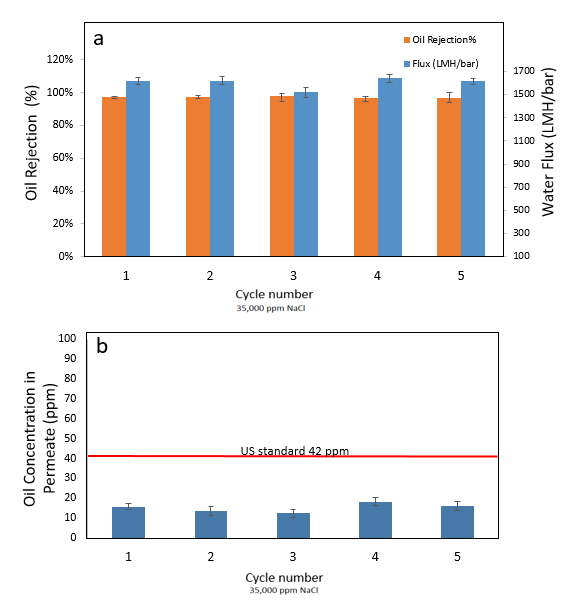


Figure S8: Reusability test of the TNFS membrane. a) Oil rejection and water flux as a function of 5 cycles of reuses, b) oil concentration in the permeate as a function of 5 cycles of reuses.


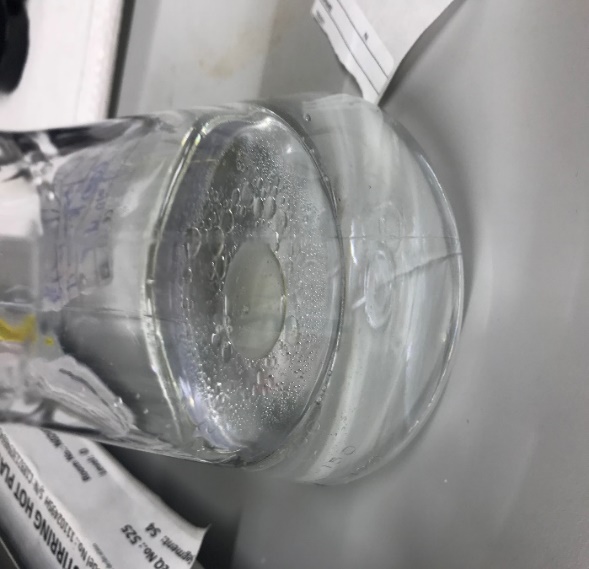

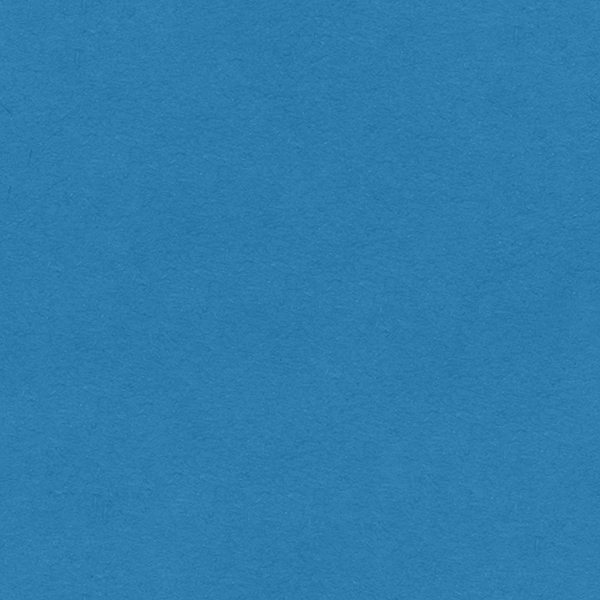


After sonication: one phase

Before sonication: Two phases
